# Evolution of SARS-CoV-2 in Spain during the First Two Years of the Pandemic: Circulating Variants, Amino Acid Conservation, and Genetic Variability in Structural, Non-Structural, and Accessory Proteins

**DOI:** 10.3390/ijms23126394

**Published:** 2022-06-07

**Authors:** Paloma Troyano-Hernáez, Roberto Reinosa, África Holguín

**Affiliations:** HIV-1 Molecular Epidemiology Laboratory, Microbiology Department and Instituto Ramón y Cajal de Investigación Sanitaria (IRYCIS) in Hospital Universitario Ramón y Cajal, CIBER en Epidemiología y Salud Pública (CIBERESP), Red en Investigación Translacional en Infecciones Pediátricas (RITIP), 28034 Madrid, Spain; troyanopaloma@gmail.com (P.T.-H.); roberto117343@gmail.com (R.R.)

**Keywords:** SARS-CoV-2, Spain, lineages, non-structural proteins, accessory proteins, structural proteins, epidemic waves, variability, mutation frequency

## Abstract

Monitoring SARS-CoV-2’s genetic diversity and emerging mutations in this ongoing pandemic is crucial to understanding its evolution and ensuring the performance of COVID-19 diagnostic tests, vaccines, and therapies. Spain has been one of the main epicenters of COVID-19, reaching the highest number of cases and deaths per 100,000 population in Europe at the beginning of the pandemic. This study aims to investigate the epidemiology of SARS-CoV-2 in Spain and its 18 Autonomous Communities across the six epidemic waves established from February 2020 to January 2022. We report on the circulating SARS-CoV-2 variants in each epidemic wave and Spanish region and analyze the mutation frequency, amino acid (aa) conservation, and most frequent aa changes across each structural/non-structural/accessory viral protein among the Spanish sequences deposited in the GISAID database during the study period. The overall SARS-CoV-2 mutation frequency was 1.24 × 10^−5^. The aa conservation was >99% in the three types of protein, being non-structural the most conserved. Accessory proteins had more variable positions, while structural proteins presented more aa changes per sequence. Six main lineages spread successfully in Spain from 2020 to 2022. The presented data provide an insight into the SARS-CoV-2 circulation and genetic variability in Spain during the first two years of the pandemic.

## 1. Introduction

Coronavirus disease (COVID-19) was detected for the first time in December 2019 in Wuhan, China [1]. In January 2020, the responsible virus, acute respiratory syndrome coronavirus 2 (SARS-CoV-2), was isolated and the complete viral genome was sequenced [2]. During the period covered in this study (February 2020 to January 2022), 10,122,981 confirmed COVID-19 cases and 93,839 deaths were declared in Spain, according to the Spanish Ministry of Health [3]. The first Spanish COVID-19 case emerged in late January 2020 [4]. However, this cannot be considered patient zero, since there were many independent SARS-CoV-2 introductions to the country at the beginning of the outbreak, with different successful lineages, probably favored by super-spreaders [4,5]. On 14 March 2020, the Spanish government implemented a national lockdown and a state of emergency until 21 June 2020 [6,7]. However, the national deconfinement plan started on 28 April 2020. This plan included four phases, with different degrees of restrictions for each Autonomous Community (AC) and phase. To contain the vast second wave of infection, during October 2020, a second state of emergency was implemented, affecting the national territory or certain ACs [8]. The last state of emergency was established on 25 October 2020 and was extended until 9 May 2021 [9,10]. To date, there have been six waves of infection in Spain [3].

SARS-CoV-2 is a ß-coronavirus belonging to the Coronaviridae family, order Nidovirales, subfamily Orthocoronavirinae. Coronaviruses (CoVs) are enveloped positive-sense RNA viruses with a large and non-segmented genome of ∼30 kb length [11]. Around two-thirds of the genome is occupied by the first two overlapping ORFs (ORF1a and ORF1b) located at the 5′ end of the viral RNA, which encode the non-structural proteins (nsps) [12,13,14]. ORF1a/b translate to a short polyprotein (pp1a) that includes nsp1–11, or a longer polyprotein (pp1ab) that includes nsp1–10 and 12–16, depending on whether the stop codon at the end of ORF1a is recognized or bypassed [12,13,15]. These polyproteins are then proteolytically processed into the 16 individual nsp by viral proteases, such as the main protease or chymotrypsin-like cysteine protease (3CLpro) and papain-like protease (PLpro) [11,13,15]. The CoVs 3′ end of the viral genome encodes the four main structural proteins—Spike (S), Envelope (E), Membrane (M), and Nucleocapsid (N)—required for the structurally complete viral particle [14], and the accessory proteins, involved in pathogenicity [16]. All Orthocoronavirinae share the four structural proteins. N forms a helical capsid that contains the genome, which is surrounded by the envelope containing the E and M proteins, while the S protein mediates viral entry into the host cells [17]. Structural and accessory proteins are synthesized from their respective subgenomic mRNAs by the replication and transcription complex (RTC). The RTC is composed of several proteins, including the RNA-dependent RNA polymerase (RdRp) (nsp12), a helicase (nsp13), an exoribonuclease (ExoN) (nsp14), processivity factors (nsp7–8), single-strand binding protein (nsp9), other cofactors such as nsp10, and capping enzymes such as nsp16 [15]. Regarding SARS-CoV-2 accessory proteins, different studies provide different annotations depending on the studied sequence [16,18,19,20]. This study considers the accessory proteins described in the NCBI reference SARS-CoV-2 sequence NC 045512.2 3a, 6, 7a, 7b, 8, and 10. The SARS-CoV-2 proteins’ proposed functions are summarized in Table 1.

The SARS-CoV-2 genome presents high homology to other human and bat CoVs, sharing around 89% sequence identity [2,13,19,96], showing higher homology with related bat-derived CoVs (88%) than with SARS-CoV (79%) or MERS-CoV (50%) [97]. Although RNA viruses have mutation rates up to a million times higher than their hosts, correlated with enhanced virulence and viral evolution capacity [98], CoVs have genetic proofreading mechanisms (ExoN) absent in other RNA viruses that limit their mutation rate [99,100], estimated at around 6 × 10^−4^ nucleotides/genome/year [96]. CoVs can also recombine through homologous and non-homologous recombination [101], which may be related to CoVs’ capacity for interspecies jumping [102]. Therefore, it is essential to monitor SARS-CoV-2’s genetic variability in this ongoing pandemic to understand its molecular evolution and ensure the performance of developing diagnostic tools, vaccines, and immunotherapeutic interventions against COVID-19.

A large number of SARS-CoV-2 variants have emerged since the beginning of the pandemic. The World Health Organization (WHO) designates some SARS-CoV-2 variants as variants of interest (VOI) or variants of concern (VOC) according to the impact of their genetic changes in the virus characteristics, course of the disease, and public health impact [103]. The currently designated VOC by the WHO are the Alpha, Beta, Gamma, Delta, and Omicron variants [103], corresponding to B.1.1.7, B.1.351, P.1, B.1.617.2, and B.1.1.529 lineages, respectively, according to Pango nomenclature [104,105,106]. Due to the wide spread of the Delta variant, the classification of this lineage was modified, breaking up B.1.617.2 into smaller clusters, the AY lineages, that are geographically related or associated with a significant epidemiological event [107]. As for the Omicron variant, two genetically distinct sublineages of B.1.1.529 have been identified to date, BA.1 and BA.2 [107] (https://www.pango.network (accessed on 9 May 2022)), being both sublineages considered Omicron VOC by the WHO [108].

The present report analyzes the epidemiology of SARS-CoV-2 in Spain by epidemiological weeks (epiweeks) and across six study periods established from the beginning of the pandemic to the sixth epidemiologic wave, using all available SARS-CoV-2 GISAID sequences collected in all regions in Spain (17 AC and 2 Autonomous Cities) during the study period. We present the circulating SARS-CoV-2 variants, the conservation and mutation rates, and the most frequent aa changes across each structural, non-structural, and accessory viral protein in each Spanish region and period.

## 2. Results

A total of 88,248 Spanish SARS-CoV-2 complete and partial sequences collected from 24 February 2020 to 29 January 2022 (corresponding to 101 epiweeks) were downloaded from the GISAID database. After discarding those undated and/or incorrectly classified, more than 70,000 sequences for each SARS-CoV-2 protein were included in the study. The number of sequences available for each protein, period, and AC are described in Supplementary Table S1.

### 2.1. Nucleotide and Amino Acid Variability in the 26 Spanish SARS-CoV-2 Studied Proteins

Nucleotide substitutions between natural bases (guanine, adenine, cytosine, and thymine) were analyzed for each of the 26 SARS-CoV-2 proteins, revealing a total of 32,334 instances of polymorphisms across the SARS-CoV-2 Spanish genomes with available sequences in the GISAID database (Table 2).

Of the total instances of polymorphisms, 17,014 (52.6%) involved transition mutations and 15,320 (47.4%) transversion mutations (Supplementary Table S2), with a ratio of 1:0.90. The group of structural proteins presented more transversion than transition events (1:2.26). All the SARS-CoV-2 genes showed more transition than transversion events among the total instances of polymorphisms, except for the nsp12 (RNA polymerase) gene with a ratio of 1:1, and for nsp11, the Spike, the Nucleocapsid, and ORF7a genes with more transversions than transitions.

**Figure 1:**
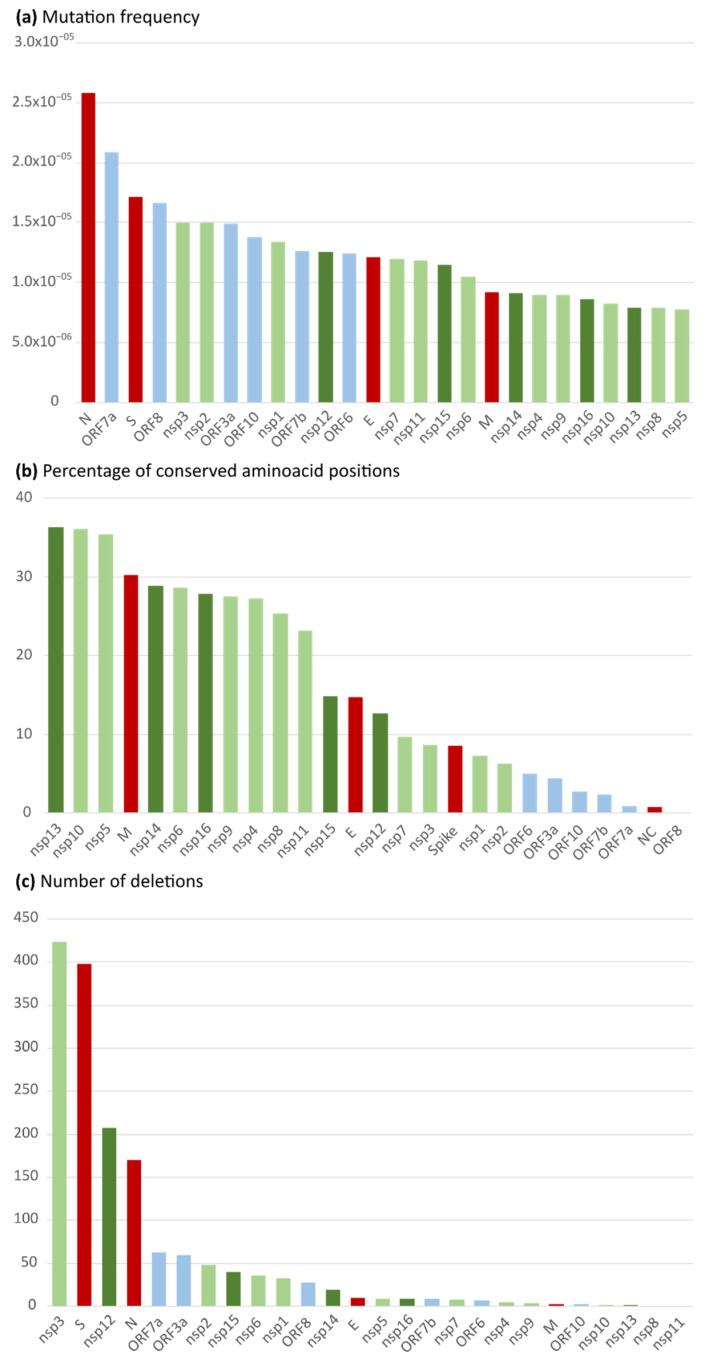
Spanish SARS-CoV-2 mutation frequency and rate of conserved aa positions per viral protein sorted from greatest to lowest. (**a**) Mutation frequency. *X* axis: mutation frequency [Mf = P i/(L n × N s)]; *Y* axis: SARS-CoV-2 loci. (**b**) Percentage of conserved amino acid positions. *X* axis: percentage of completely conserved aa sites; *Y* axis: SARS-CoV-2 proteins. (**c**) Number of total deletions in each SARS-CoV-2 protein. *X* axis: number of deletions detected; *Y* axis: SARS-CoV-2 proteins. Color code: in green: non-structural proteins, light green: ORF1ab (nsp1 to 11), dark green: ORF1b (nsp12 to 16); in blue: accessory proteins (3a to 10); in red: structural proteins. E: Envelope; M: Membrane; N: Nucleocapsid; S: Spike; nsp: non-structural protein.

The mean mutation frequency considering all the protein genomes was 1.24 × 10^−5^ (Table 2). The Nucleocapsid gene followed by ORF7a were the most mutation-prone genes (2.58 × 10^−5^ and 2.08 × 10^−5^, respectively). When comparing non-structural, structural, and accessory proteins, the mutation frequency was slightly higher in structural proteins (1.60 × 10^−5^), followed by accessory proteins (1.52 × 10^−5^), and non-structural proteins (1.05 × 10^−5^). Among the structural proteins, the mutation frequency was higher in the N gene, followed by the S, E, and M genes (Figure 1a).

After the translation of nt sequences encoding each 26 SARS-CoV-2 protein, we analyzed the number of aa changes, deletions, stop codons, and completely conserved positions. We also calculated the percentage of conservation and mean aa changes per sequence in each protein considering only valid codons (Table 3).

A total of 21,433 aa changes, 1579 deletions, and 593 stop codons were detected in the Spanish SARS-CoV-2 proteome, being nsp3 the protein with the highest number of deletions (423) and stop codons (134), followed by the Spike protein (397 and 123, respectively) (Figure 1c), being the largest proteins in the SARS-CoV-2 genome (nsp3,1,945 aa; S protein,1273 aa). Nsp3 encodes the papain-like protease (PLpro, domain within nsp3). In PLpro, we found five deleted residues in a total of eight sequences. The PLpro main catalytic residues, C111, H272, and D286 [109], were highly conserved, finding only two aa changes: C111Y in one sequence and D286N in three sequences of the total Spanish dataset.

The mean aa conservation was above 98% in all the analyzed proteins, with a global aa conservation of 99.69%, showing an average of 1.15 aa changes/deletions per sequence. The protein with the highest mean aa change/deletion frequency per sequence was the Spike (10.8), followed by the Nucleocapsid (3.79). The proteome’s mean rate of variable aa positions was 84.06%. Variable aa positions ranged from 63.73% in nsp13 to 100% in ORF8. Twelve proteins presented more than 90% variable positions along their sequence: structural proteins N and S, non-structural proteins nsp1, 2, 3, and 7, and all accessory proteins (ORF 3a-10). The percentage of conserved positions for each protein is illustrated in Figure 1b.

Although the mutation frequency (Mf) and mean aa conservation were similar between the three groups of studied proteins, non-structural proteins presented the lowest mutation frequency (1.05 × 10^−5^) and percentage of variable aa positions (79.19%) and the highest aa conservation (99.84%). Structural proteins presented the highest mutation frequency (1.60 × 10^−5^) and mean aa changes/deletions per sequence (3.87). Meanwhile, accessory proteins showed the greatest percentage of variable aa positions (99.36%) and the lowest aa conservation (97.49%), but also the lowest number of aa changes/deletions per sequence (0.68). Among the structural proteins, the Nucleocapsid protein presented a higher rate of variable positions, followed by the Spike, the Envelope, and the Membrane proteins (Table 3). Our data revealed that nsp13 and nsp10 were the SARS-CoV-2 proteins with the lowest percentage of variable positions (63.73% and 64.03%, respectively), and N and ORF8 the highest, being 99.28% and 100%, respectively (Table 3). Within the Spike, the receptor-binding domain (RBD) region had a mean conservation of 98.89%, with 2.4 mean changes per sequence. All the aa changes detected in this region had a frequency below 10%, except for L452, T478, and N501, the three of them located in the receptor-binding motif.

The residues of the SARS-CoV-2 main protease (nsp5) involved in binding for remdesivir and Paxlovid (two antivirals recommended by the WHO for COVID-19 treatment in patients at risk of hospital admission) were highly conserved in our sequence dataset, with a percentage of mutated sequences below 0.2%. Some of these residues are involved in the binding of both drugs (C145, E166, H163, H164, Q192), while G143 is involved in Paxlovid binding, and other sites interact with remdesivir (H41, M49, Y54, F140, N142, S144, M165, L167, P168, H172, N187, R188, Q189, T190, and A191) [110,111]. We only found one sequence with a deletion in residue 192 (involved in the binding of both drugs) and no other aa changes in the rest of the sites that interact with Paxlovid, which showed complete conservation among the whole Spanish sequence dataset. As for other nsp5 residues that interact with remdesivir, we found aa changes in eight of them (H41Q, M49I/V, N142S, M165I, P168S, R188K/S, Q189K, and A191V/S). A191 was the most frequently mutated residue, presenting changes in 96 sequences, but with an overall very low variability frequency (0.11%). The main change was A191V (85 sequences), followed by A191S (11 sequences) and M49I (9 sequences). The rest of the changes appeared in less than five sequences of the complete dataset.

### 2.2. Amino Acid Variability in Spanish SARS-CoV-2 Structural Proteins

**Figure 2:**
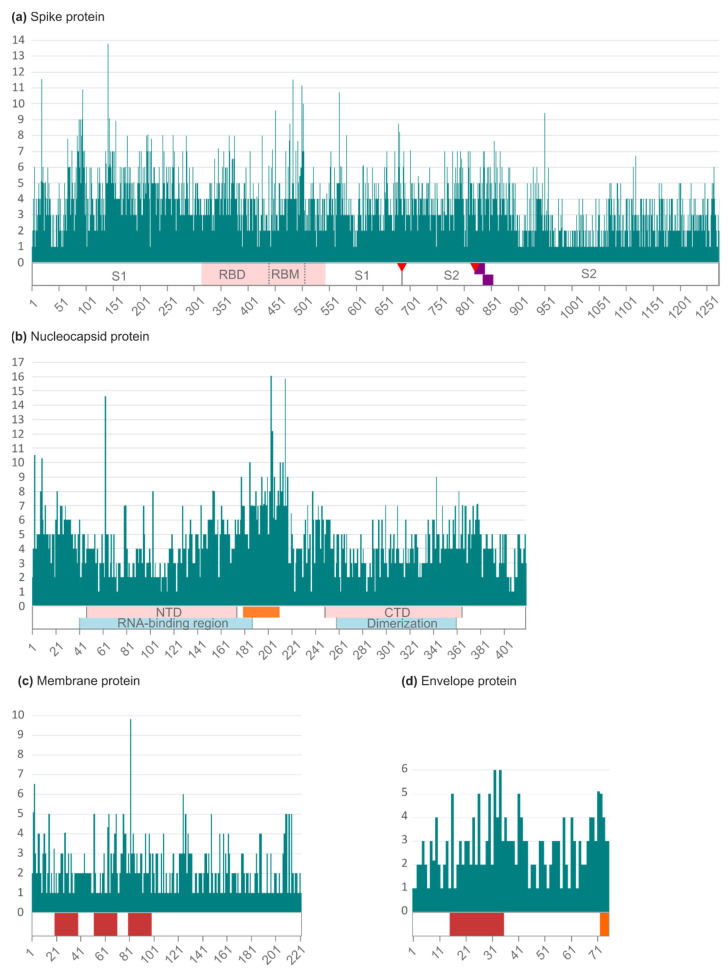
SARS-CoV-2 structural proteins’ Wu–Kabat variability coefficient plot and main protein regions. *Y*-axis: variability coefficient. *X*-axis: amino acid position and main protein domains. (**a**) Spike protein; RBD: receptor-binding domain; RBM: receptor-binding motif; red triangles: cleavage sites S1/S2 and S2′; purple boxes: fusion peptides 1 and 2. (**b**) Nucleocapsid protein; NTD: N-terminal domain; CTD: C-terminal domain; orange box: SR-rich linker. (**c**) Membrane protein; red boxes: transmembrane domains. (**d**) Envelope protein; red box: transmembrane domain; orange box: PDM (PDZ-binding motif).

The Wu–Kabat protein variability coefficient (WK) was analyzed in the four structural proteins to study the susceptibility of an aa position to evolutionary replacements (Supplementary Table S3). The analysis showed the position-specific aa variations according to their frequency in the structural proteins and their main domains (Figure 2).

In the Spike protein (Figure 2a), 10.37% of its positions (132 among 1273 aa) had a WK of 1 among the 83,928 analyzed sequences. The maximum coefficient was 13.79, found in position 142 (G142V/S/D/Y/C/N/M/F), located in the Spike S1 subunit. The protein’s cleavage site 1 (residue 685) had a WK of 4 with the changes R685S/F/H, while cleavage site 2 (residue 815) showed a coefficient of 5 with the changes R815K/G/H/N. The receptor-binding domain (223 aa) within the S protein had a median WK of 4 with a maximum coefficient of 11.53 in site 484 (E484A/K/Q/G/S/V/L/D/M), followed by site 501 (WK 11.13, N501S/Y/T/I/H/K). These last two sites (484 and 501) are located within the receptor-binding motif (aa 437–508). The S2 subunit (588 aa) showed less variability than S1 (672 aa), with a mean Wu–Kabat coefficient of 3 vs. 4, and 17.86% of its sites with a WK of 1 vs. 3.57% in S1.

In the Nucleocapsid (Figure 2b), the highest aa variability coefficient was 16.03 in site 203 (R203K/M/S/Q/V/T/I/E), located within the serine/arginine-rich (SR) linker (positions 180–210). This region showed a median WK of 7, being 203 the site with the higher WK, followed by site 204 (WK 12.19, G204R/L/V/P/Q/A/E/I). The RNA-binding domain (146 aa) and the dimerization domain (104 aa) had a median WK of 4. A WK of 1 was found in 3.10% of the total 419 sites of the Nucleocapsid protein, 3.42% residues of the RNA-binding domain (146 aa), 2.88% residues of the dimerization domain (104 aa), and none (WK 0%) in the SR linker.

In the Membrane protein (Figure 2c), 31.53% of the 222 sites had a WK of 1, with a maximum aa variability of 9.83 in site 82 (I82F/T/S/V), located in the third transmembrane domain. The sites located on the surface had a slightly lower WK median (WK 3) than the transmembrane and intravirion sites (WK 2) and fewer positions with a WK of 1 (19.23% vs. 23.81% and 37.88%, respectively).

The Envelope protein had a Wu–Kabat coefficient of 1 in 16% of its 75 residues (Figure 2d). The maximum WK was 6 in positions 32 (A32G/R/I/E/P) and 34 (L34Y/A/F/H/T), located in the transmembrane domain. The rate of sites with a coefficient of 1 was higher in the surface domain (30.77%), followed by the intravirion domain (14.63%), and the transmembrane domain (9.52%). The PDZ-binding domain (positions 72–75) had a median Wu–Kabat coefficient of 3.5, with all its residues presenting a WK > 1.

### 2.3. Most Prevalent aa Changes and Deletions in the Spanish Sequences

**Figure 3:**
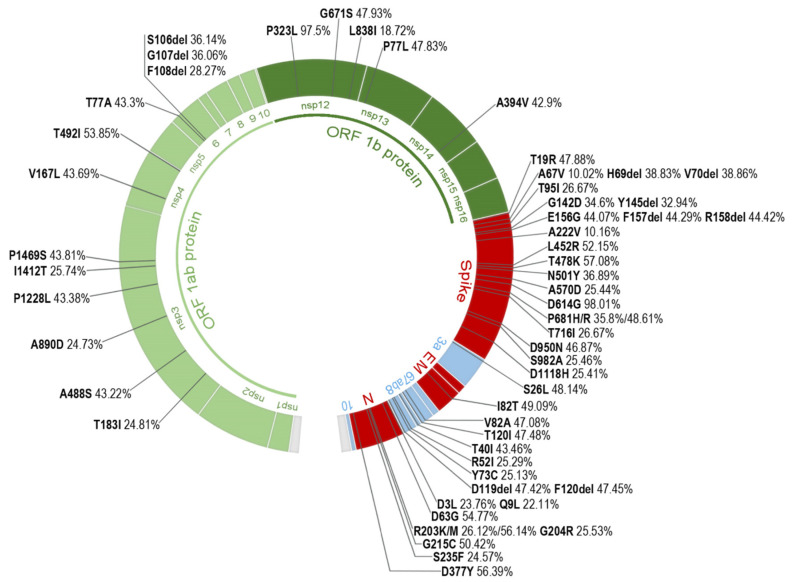
Amino acid changes and deletions present in ≥10% of the Spanish SARS-CoV-2 sequences. Color code: in green—non-structural proteins, light green: ORF1ab nsp1 to 11, dark green: ORF1b nsp12 to 16; in red—structural proteins; in blue—accessory proteins 3a to 10. E: Envelope, M: Membrane, N: Nucleocapsid; nsp: non-structural protein; del, deletion.

We identified all aa changes and deletions present in ≥10% of the total Spanish sequences per protein in the whole SARS-CoV-2 sequence set, finding 57 changes present in 13 proteins: non-structural proteins 3, 4, 6, 12, 13, and 14; structural proteins Spike, Membrane, and Nucleocapsid; and accessory proteins 3a, 7a, 7b, and 8. Their locations in the genome and prevalence are described in Figure 3. More than half of these changes (56%) were located in structural proteins, 30% were found in nsp, and 14% in accessory proteins. The Spike protein presented the greater number of changes present in ≥10% of the sequences (22), followed by the Nucleocapsid (9). The most frequent aa change in the Spanish dataset was D614G (98.01%) in the Spike protein, followed by P323L (97.50%) in nsp12 and T478K (57.08%) also in the Spike protein.

Most of the other 13 proteins showed a low frequency in their most prevalent aa changes among the available Spanish sequences during the study period: seven of them under 1% (nsp7, nsp8, nsp10, nsp11, nsp15, nsp16, ORF6), and two of them below 2% (nsp1 and nsp9). Four proteins presented changes slightly more prevalent among their sequences: V485I in nsp2 (5.13%), V30L in ORF10 (6.12%), P132H in nsp5 (8.19%), and T9I in E (8.22%). All aa changes, deletions, and stop codons found in each protein and their frequency are described in Supplementary Table S4.

**Figure 4:**
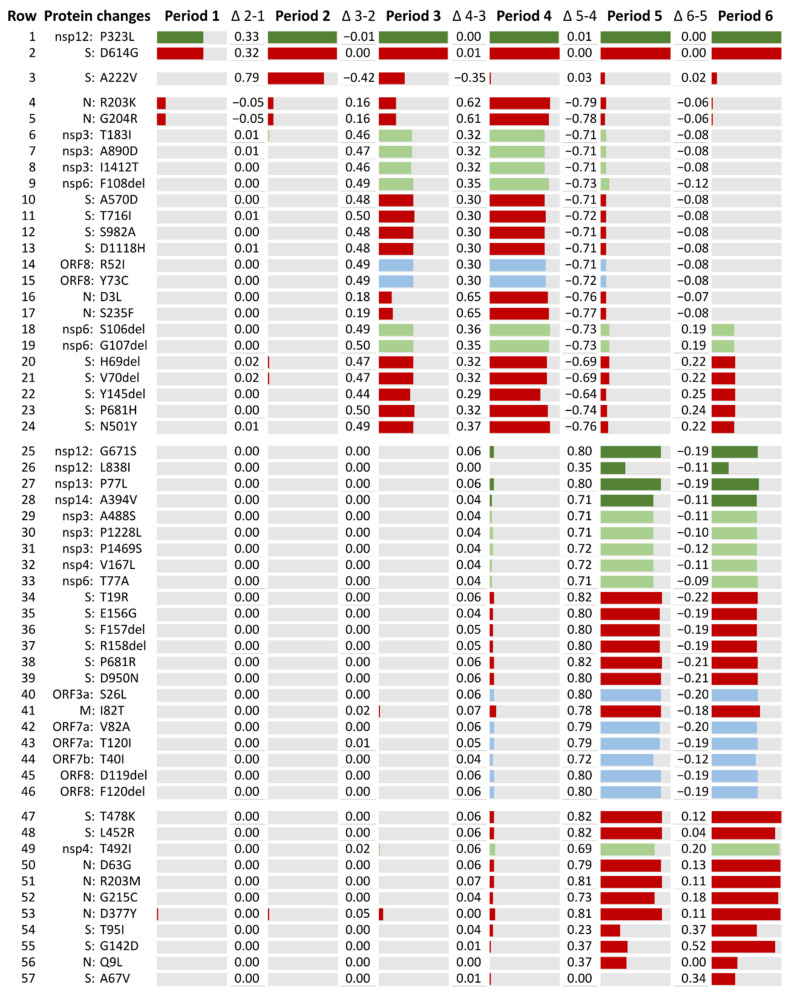
Frequency difference (Δ) of the 57 amino acid changes and deletions present in ≥10% of the Spanish SARS-CoV-2 sequences over the six waves according to the Spanish epidemic curve. Under “Protein changes” heading: protein and aa change present in ≥10% of the Spanish sequences; E: Envelope, M: Membrane, N: Nucleocapsid, S: Spike, nsp: non-structural protein; colored bars: frequency of the aa change for each study period. In green: non-structural proteins, light green: ORF1ab nsp1 to 11, dark green: ORF1b nsp12 to 16; in red: structural proteins; in blue: accessory proteins 3a to 10. Period 1: epiweeks 2020.9 to 2020.25. Period 2: epiweeks 2020.26 to 2020.49. Period 3: epiweeks 2020.50 to 2021.10. Period 4: epiweeks 2021.11 to 2021.24. Period 5: epiweeks 24.2021 to 41.2021. Period 6: epiweeks 42.2021 to 4.2022. Δ: frequency difference between periods. Positive Δ values indicate an increase in the aa change frequency, negative Δ values indicate a decrease in the aa change frequency, and Δ values close to zero indicate no or minimal frequency change.

The frequency of these 57 changes and deletions was further analyzed by epiweeks, grouped into the six previously described periods, in line with the Spanish epidemic curve: 1 (24 February to 20 June 2020), 2 (21 June to 5 December 2020), 3 (6 December 2020 to 13 March 2021), 4 (14 March to 19 June 2021), 5 (20 June to 16 October 2021), and 6 (17 October to 29 January 2022). According to the frequency difference (Δ) of these aa changes between consecutive periods, the aa substitutions were grouped into five categories (Figure 4). 

In the first category (rows 1–2 in Figure 4), we grouped the aa changes that became predominant early in the pandemic and fixated in the genome, being present in >99% of the sequences in the following periods: D614G in the Spike protein and P323L in nsp12. Both changes could be detected since period 1.2. The second category (row 3) had Spike’s aa change A222V, which increased only in period B (80% of the Spike’s sequences) but decreased in the following periods. The third category (rows 4–24) lists the 21 aa changes that increased their frequency during the third wave or period 3, with a maximum prevalence during period 4, decreasing in the next period. Within this group, seven changes (2 in nsp6 and 5 in the Spike) show an increasing tendency in the last period of this study. The fourth category (rows 25–46) corresponds to the 22 aa changes that increased in period 5, slightly decreasing their frequency in the next epidemic wave. Finally, the last category (rows 47–57) shows the aa changes that became more frequent in the last period.

**Figure 5:**
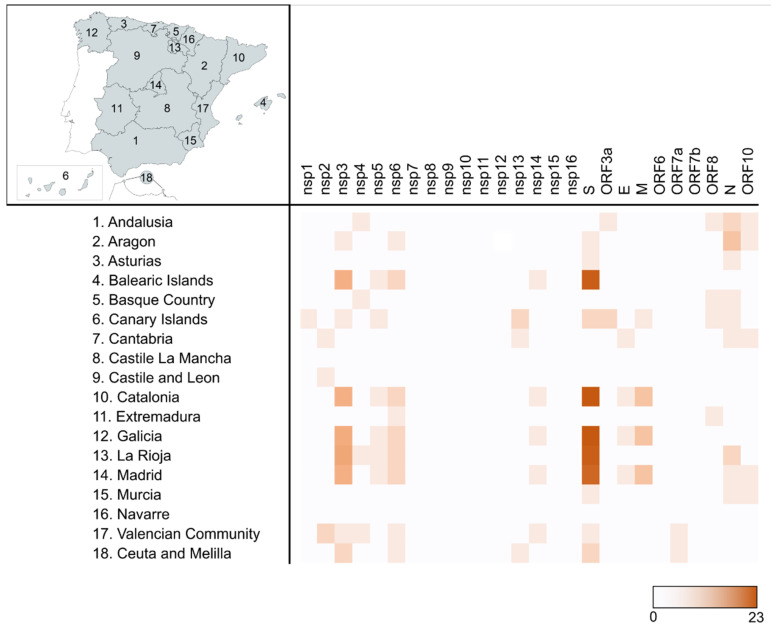
Number of aa changes and deletions different from those reported in Figure 3 and present in ≥10% of SARS-CoV-2 sequences from the Spanish Autonomous Communities. Autonomous Communities: 1–17 in the map; Autonomous Cities (Ceuta and Melilla): number 18 in the map. Nsp: non-structural protein, S: Spike protein, E: Envelope protein, M: Membrane protein, N: Nucleocapsid protein.

To detect aa changes or deletions with a significant prevalence in the AC, regardless of their frequency in the total Spanish available sequences during the study period, changes with a frequency ≥10% in each of the 18 AC were analyzed. After discarding the changes that coincided with the 57 most frequent aa changes previously described in the complete set of Spanish sequences (Figure 3), 79 additional changes were found (Supplementary Table S5). Most of these changes were located in the Spike (30). The AC harboring the highest number of changes was Galicia (36), followed by Catalonia (35), and La Rioja and Madrid (34) (Figure 5). Two AC, Castile La Mancha and Navarre, showed no additional changes. Among the AC, most of these changes were present in the Spike, nsp3, nsp6, and the Nucleocapsid.

A total of 29 aa changes were found in five or more AC: four in nsp3 (K38R, S1265del, L1266I, and A1892T), one in nsp5 (P132H), two in nsp6 (L105del and I189V), twenty in the S protein (V143del, Y144del, D796Y, E484A, G339D, S371L, S373P, S375F, S477N, Q493R, G496S, Q498R, Y505H, T547K, H655Y, N679K, N856K, Q954H, N969K, and L981F), one in ORF10 (V30L), and one in the N protein (A220V). The latter was the change found in the largest number of AC (8): Andalusia, Aragon, Asturias, Cantabria, Canary Islands, Murcia, Madrid, and La Rioja, being present in 7.44% of the total Spanish sequences.

Three aa changes were present with a frequency ≥25% in at least one AC: nsp13 K460R in Cantabria (33.13%), ORF10 V30L in Aragon (28.10%), and Nucleocapsid A220V in Aragon and Madrid (31.80% and 26.18%, respectively). These changes were further analyzed to detect if they could be allocated to one or more periods among the six studied. K460R was present throughout the third period and first half of the fourth. V30L was detected mainly in period 2, although it persisted until period 4. A220V was detected earlier in Madrid, in period 1.2, while in Aragon, its detection was delayed until period 2.1, although it reached a greater frequency during the third period in both AC.

### 2.4. SARS-CoV-2 Lineages Circulating in Spain during the First Year of the Pandemic per Study Period

**Figure 6:**
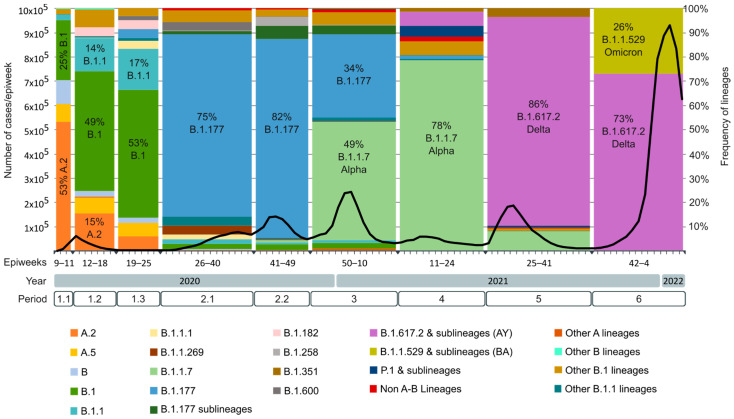
Epidemic curve and main SARS-CoV-2 lineages circulating in Spain per study period. The bold black line represents the epidemic curve with the number of SARS-CoV-2 cases per epidemiological week according to the official data available from the Spanish National Epidemiological Surveillance Network (RENAVE, https://cnecovid.isciii.es/covid19 (accessed on 7 April 2022)). Study period dates according to Table 2.

After performing the sequence quality control as described in the Methods section, a total of 82,655 sequences were successfully assigned to a lineage according to the Pangolin COVID-19 Lineage Assigner. The complete classification is available in Supplementary Table S6. Figure 6 illustrates the main SARS-CoV-2 lineages per period in Spain after analyzing all available Spanish sequences deposited in GISAID. The figure also includes the epidemiological curve according to the RENAVE Spanish COVID-19 incidence data for each epidemiological week. The Spanish incidence and mortality information retrieved from RENAVE in the study period can be found in Supplementary Table S7 and Figure S1.

During the first study period (24 February to 20 June 2020), before the national lockdown (period 1.1, 24 February to 14 March 2020), a total of 11 lineages were circulating in Spain among the sequences available in GISAID. A lineages predominated over B lineages (60.49% vs. 39.51%), with 53.09% of the sequences belonging to lineage A.2 and 7.28% to lineage A.5. After the lockdown, the presence of B lineages increased to 77.86% before the deconfinement plan (period 1.2, 15 March to 2 May 2020), and to 88.73% until the end of the first state of alarm (period 1.3, 3 May to 20 June 2020), being B.1 the most successful lineage in both periods. The diversity of lineages increased in period 1.2 (39 lineages detected), but during the confinement (period 1.3), the diversity decreased again (17 lineages).

During the second study period (21 June to 5 December 2020), the most successful lineage circulating in Spain was B.1.177. In period 2.1 (21 June to 3 October 2020), 75.25% of the sequences belonged to the B.1.177 lineage. Of the 44 total lineages detected in this period, 14 of them were B.1.177 descendants. The first nonA-nonB lineages in Spain were detected in this period in seven sequences (C.21, C.35, and N.2, all European lineages). In period 2.2 (4 October to 5 December 2020), 82.17 % of the sequences belonged to the B.1.177 lineage, being 12 of the 35 detected lineages B.1.177 descendants. In this period, the B.1.1.7 VOC (Alpha variant) was detected for the first time in eight sequences (0.78%) collected in the Valencian Community. Another two nonA-nonB lineages were detected in four sequences (C.36, mainly Egyptian, and W.1, related to France and the US).

In the third period (6 December 2020 to 13 March 2021), the B.1.177 lineage’s frequency decreased to 34.29%, with the B.1.1.7 VOC (Alpha variant) becoming the most successful lineage, representing 48.99% of the total sequences. Of the 98 lineages detected, nine were nonA-nonB lineages related to European and non-European countries, including the P.1 VOC (Gamma variant), detected in 15 sequences (0.2%). The B.1.351 VOC (Beta variant) was also detected in this period in 31 sequences (0.4%).

In the fourth period (14 March 2021 to 19 June 2021), the B.1.1.7 VOC remained the main circulating variant in Spain, representing 78% of the sequences. The Gamma and Beta VOC increased slightly their frequency (4.44% and 1.61%, respectively), but remained a minority. The XB recombinant was also detected in this period in 10 sequences. This was the first period where the Delta VOC (B.1.617.2 and AY sublineages) was detected. Among the 150 lineages and sublineages detected in this period, 25 belonged to the Delta VOC. Although it represented only 5.74% of period 4 sequences, during the next period, it became the main circulating variant, increasing its frequency to 86.10%, while B.1.1.7’s prevalence decreased to 7.95%. Almost half of the lineages and sublineages detected in this period were Delta sublineages (112/146). The Gamma and Beta VOC could still be detected in period 5 in low frequency (0.71% and 3.69%, respectively). In the last study period or period 6, the Delta VOC remained the most frequent variant (72.98%). Delta sublineages represented 74% of the circulating lineages and sublineages during this period (127). The Omicron VOC was introduced and quickly increased its frequency over the epidemiological weeks, representing 26.99% of the sequences circulating in Spain in period 6.

**Figure 7:**
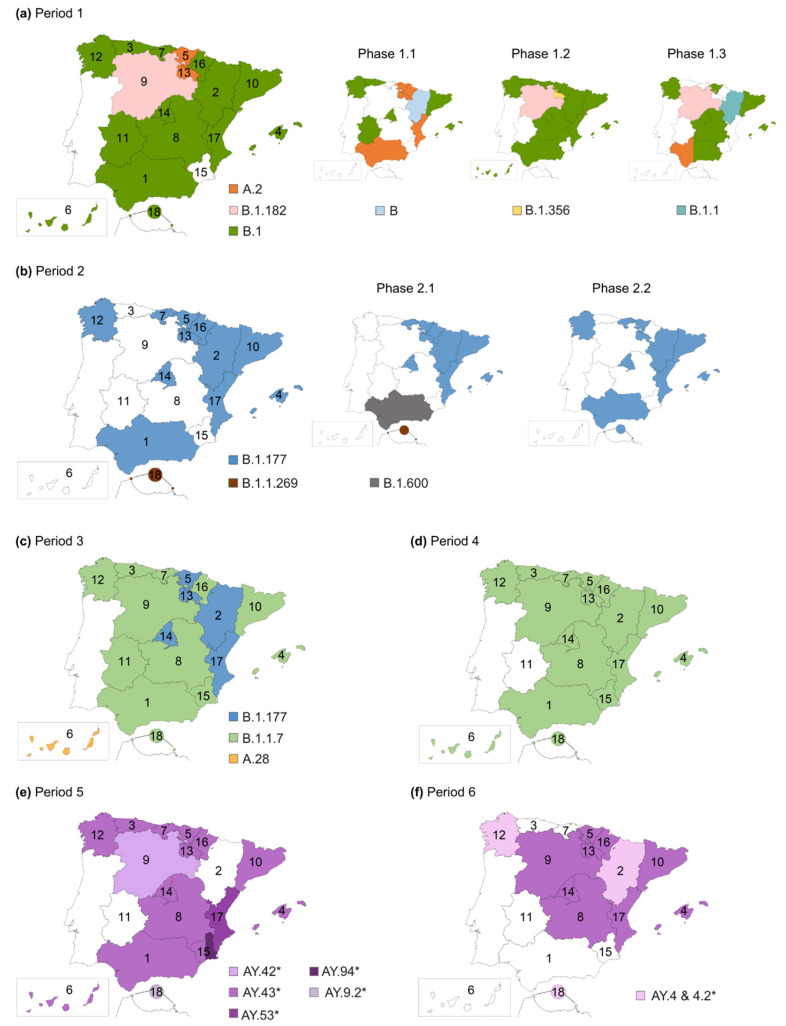
Main SARS-CoV-2 lineages in the Spanish Autonomous Communities with more than 10 sequences available in GISAID for each study period. (**a**) Period 1. (**b**) Period 2. (**c**) Period 3. (**d**) Period 4. (**e**) Period 5. (**f**) Period 6. In color: AC with more than 10 sequences available in GISAID for each period. 1: Andalusia 2: Aragon, 3: Asturias, 4: Balearic Islands, 5: Basque Country, 6: Canary Islands, 7: Cantabria, 8: Castile La Mancha, 9: Castile and Leon, 10: Catalonia, 11: Extremadura, 12: Galicia, 13: La Rioja, 14: Madrid, 15: Murcia, 16: Navarre, 17: Valencian Community, 18: Ceuta and Melilla). Study period dates according to Table 2. B.1.1.7 (Alpha variant), B.1.351 (Beta variant), P.1 (Gamma variant), B.1.617.2/AY (Delta variant). *, the clusters of the Delta variant.

The number of available SARS-CoV-2 sequences in each AC was uneven (Supplementary Table S6). Figure 7 illustrates the SARS-CoV-2 lineages’ evolution in each Spanish AC and study period, after including the AC with at least 10 sequences for each phase or period.

In period 1.1, the A.2 lineage predominated in Andalusia, Basque Country, La Rioja, Navarre, and the Valencian Community, whereas B lineages (B.1 followed by B) were the main circulating lineages in Aragon, Asturias, Balearic Islands, Catalonia, Extremadura, Galicia, and Madrid (Figure 7). In the next period (1.2), B.1 predominated in all AC, except for Castile and Leon, where B.1.182 (another mainly Spanish lineage) was the main lineage, and La Rioja, where most sequences belonged to the B.1.356 lineage, a European lineage mostly Spanish and Dutch. In the last part of period 1, B.1 was still the main lineage, except for Castile and Leon with B.1.182 predominance, Aragon, where the B.1.1 lineage predominated, and Andalusia, where the same number of sequences belonged to the B.1 and A.2 lineages. In this period, B.1.177, the main lineage in Spain during period 2, was already present in Aragon and the Balearic Islands.

Throughout period 2, B.1.177 was the most successful lineage in Spain, as previously described. However, in period 2.1, other lineages predominated in two AC: B.1.600 (lineage mainly present in Spain and Bolivia) in Andalusia, and B.1.1.269 (European lineage) in Ceuta and Melilla.

In period 3, the B.1.1.7 VOC (Alpha variant) became the predominant variant in most AC, except for the Canary Islands, where A.28 was the main variant, and five AC where B.1.177 remained the main variant (Aragon, Basque Country, La Rioja, Madrid, and Valencian Community). However, in the Basque Country, Madrid, and the Valencian Community, the Alpha variant was present in more than 30% of their sequences. Although less frequent, the P.1 VOC (Gamma variant) and B.1.351 VOC (Beta variant) were detected in several AC (Supplementary Table S6), P.1 mainly in the Valencian Community, Catalonia, and Madrid, and B.1.351 in Catalonia.

In period 4, the Alpha VOC (B.1.1.7) became the main variant in all the Spanish AC. The Delta variant (B.1.617.2/AY), the main circulating variant in the subsequent periods, was detected in 11 AC: Asturias, the Balearic Islands, Basque Country, Castile La Mancha, Castile and Leon, Catalonia, Galicia, Madrid, Murcia, Navarra, and the Valencian Community. The main Delta clusters detected in period 4 were AY.53 (mainly a Spanish subclade), primarily detected in Madrid, Catalonia, and the Valencian Community; AY.71 (cluster mainly present in Italy, Germany, and Turkey) in Asturias and the Balearic Islands; and AY.5 (mainly a United Kingdom subclade), in Catalonia, Madrid, and Castile La Mancha.

In the next two periods, the Delta variant (in purple in Figure 7) was the main variant in all the Spanish AC. During period 5, the major Delta clusters were AY.43 (cluster mainly present in Germany, France, and United Kingdom) in most AC; AY.42 (mainly present in Germany, Spain, and France) in Castile and Leon; AY.53 in the Valencian Community; and AY.9.2 (mainly from Germany and The Netherlands) in Ceuta and Melilla. AY.94 (mainly a German cluster) shared the same number of sequences in Murcia with the AY.43 subclade. Other frequent Delta subclades were AY.4 (mainly a United Kingdom cluster) in the Balearic Islands, Castile and Leon, and Catalonia; the previously mentioned AY.5 and AY.98.1 (mainly French and German subclade) in Catalonia and Castile and Leon; AY.125 also in Catalonia (mainly from France and Germany); and AY.9.2 in Madrid and the Valencian Community.

In the last period, period 6, the main Delta subclades were AY.43 in most AC and AY.4 and AY.4.2 (clusters mainly from the United Kingdom) in Asturias, Galicia, and Ceuta and Melilla. Nevertheless, the main delta subclades in the previous period were still frequent, and other Delta sublineages became more prevalent, such as AY.119 (cluster mainly from the United States of America) in Asturias and Castile and Leon, AY.122 (from Germany and the United States of America) in Catalonia and Castile and Leon, and AY.127 (from India, the United Kingdom, and Germany) in Catalonia. The Omicron VOC could be detected in 12 of the 18 AC (Supplementary Table S6), accounting for more than 25% of the AC sequences in half of them: Castile La Mancha (27.18%), Asturias (27.32%), Balearic Islands (32.87%), Galicia (37.53%), Catalonia (39.01%), and Madrid (41.84%). The most common Omicron sublineage among the AC was BA.1.17.2 (mainly present in the United Kingdom).

## 3. Discussion

Monitoring SARS-CoV-2’s genetic diversity and emerging mutations in this ongoing pandemic is essential to understand the evolutionary trend of this new coronavirus and to ensure the performance of new diagnostic tests, vaccines, and therapies against COVID-19. Spain has been one of the European countries with the highest number of COVID-19 cases, according to the European Centre for Disease Prevention and Control (ECDC, https://www.ecdc.europa.eu (accessed on 22 January 2022)). Previous studies have analyzed the epidemiology of SARS-CoV-2 in Spain [5,112], certain Spanish cities or AC [113,114,115,116,117,118], and variants [119,120]. However, as far as we know, this is the first study including all SARS-CoV-2 GISAID available sequences from the 17 AC and 2 Autonomous Cities since the beginning of the pandemic until the sixth epidemiologic wave, including the most prevalent mutations. This descriptive study reports not only on the Spanish circulating variants in the different study periods and AC, but also on the conservation, most frequent aa changes, mutation rate, and genetic variability across structural, non-structural, and accessory SARS-CoV-2 proteins in Spain during the first two years of the pandemic, discussing their possible structural and biological implications.

In the Spanish SARS-CoV-2 sequences downloaded until January 2022, the mean genome mutation frequency (Mf) was 1.24 × 10^−5^. In a previous study with SARS-CoV-2 global sequences collected until 21 August 2020, the overall point mutations took place at a frequency of 9.4 × 10^−6^ [121]. Despite the difference regarding the geographical origin of the samples, this suggests an increase in the number of point mutations along the viral genome throughout the last few years.

The mutation frequency (Mf) and mean aa conservation were similar between non-structural, structural, and accessory proteins, while the percentage of variable positions in the aa sequence and the mean changes per sequence showed greater differences among the three groups of proteins.

Non-structural proteins had the lowest Mf (1.05 × 10^−5^), highest Ts/Tv ratio, highest conservation (99.84%), and least variable aa positions per sequence (1.25). The fact that many nsp are involved to a greater or lesser extent in the replication and transcription complex (Table 1) could explain why these proteins are more conserved and less mutation-tolerant. However, in Roy et al.’s analysis, nsp presented a much lower Mf (8.78 × 10^−6^) [121], suggesting that, although highly conserved, point mutations have increased even in non-structural proteins.

Structural proteins presented the highest Mf (1.60 × 10^−5^), being the only group of proteins with more transversion than transition events (Ts/Tv ratio 1:2.26). In Roy et al.’s study, all the SARS-CoV-2 genes had transition:transversion ratios greater than 1, although a considerable number of transversions were detected, highlighting the fact that these mutations are less likely to maintain the structural properties of the original amino acids [121]. Nevertheless, Roy et al.’s study was performed before the circulation of more heavily mutated VOC, while, in our study, there was a large proportion of sequences belonging to VOC lineages. The fact that VOC harbor a significant number of mutations in the Spike protein [122,123,124] would correlate with the greater number of mean aa changes per sequence in the structural proteins (3.87), mainly in the Spike (10.80), found in our dataset.

Despite these results, accessory proteins presented lower aa conservation and a greater number of variable aa positions compared to the structural proteins (99.36% vs. 99.42% and 97.49% vs. 86.49%, respectively), indicating that, in accessory proteins, the mutations affect more residues along the length of the protein, while, in the structural proteins, mutations are concentrated in certain positions of the protein gene. Many accessory proteins influence the host immune response and participate in viral virulence (Table 1). This fact could be related to the greater variability detected in these proteins, given that, in the context of the adaptation of SARS-CoV-2 to the human host throughout the pandemic, the virus has been progressively exposed to natural or vaccine-induced antibodies.

In *Orthocoronavirinae*, the sections of the genomes that show the largest divergence in protein domains are located in the proteins encoded in the N-terminal end of the ORF1ab, the Spike, and mainly in the accessory proteins, where each subgenus possesses an almost subgenus-specific set of accessory proteins [17]. On the other hand, the other structural proteins and the nsp implicated in the RTC, such as 3C-like protease (nsp5), RNA-dependent RNA polymerase (RdRp, nsp12), and Helicase (nsp13), show stable domain architectures across all *Orthocoronavirinae* [17]. In our Spanish sequence set, accessory proteins showed the lowest percentage of conserved positions (Figure 1b). Among them, ORF8 presented changes in all its positions. ORF8 has been described as a rapidly evolving accessory protein, proposed to interfere with immune responses [88], mediating the immune evasion of SARS-CoV-2 [86], which can explain why ORF8 harbored changes in 100% of its residues. In contrast, the proteins with the highest number of conserved positions were the nsp, specifically nsp5, nsp10, and nsp13 (>35% of conserved positions, Figure 1b).

Among the four structural proteins, the Nucleocapsid and the Spike genes showed more transversion than transition events, being N the most mutation-prone gene with the highest mutation frequency (Table 2), a trend previously observed in other studies performed in Spain and other countries, such as Canada and South Africa [125]. The Spike presented the highest mean aa change/deletion frequency per sequence, while presenting more conserved positions along its structure than N, pointing again to the high presence in the total sample of Spike heavily mutated VOC. When examining the aa conservation, it was over 99% in the four structural proteins, slightly higher in the Envelope (99.84%), which also presented fewer mean changes per sequence. The Membrane was the protein with a lower Mf (9.14 × 10^−6^), lower percentage of variable positions, and more sites with a WK of 1 (31.53% of its positions). The lower variability of M and E is in line with other studies including worldwide sequences [125,126,127]. However, the E gene has shown signatures of positive selection along with S in previous studies [128].

Analyzing the position-specific aa variability in the structural proteins can point to which regions or domains of the protein are most and least conserved. This can be useful to put into context the performance of current real-time reverse transcriptase-polymerase chain reaction (RT-PCR)-based diagnostic tests or for a more rationale design of new diagnostic tests and vaccines. The introduction of the Alpha variant revealed that the failure of some RT-PCR-based diagnostic tests to detect the S gene (S gene dropout) could be used for its diagnosis [129,130]. This method was widely used to detect this variant in Spain during the first few months after its introduction [131]. Although newer RT-PCR tests have introduced many other targets, S gene dropout could still be useful to detect the Omicron variant with some of them [132,133].

In the Wu–Kabat analysis, 132 of the Spike’s positions had a WK of 1, indicating no aa variability, most of them within the S2 subunit. Compared to a similar analysis performed on worldwide Spike sequences retrieved until June 2020 by Rahman et al., our results showed a much lower rate of invariable positions (48% vs. 10%) [134], indicating a greater number of Spike’s sites prone to aa changes in the last few years, compatible with viral evolution. However, 76% of these completely conserved sites were the same positions as in Rahman’s et al. study, most of them (86%) located in the S2 subunit. As for highly variable sites, our analysis showed a 25-times increase in sites with WK > 4 (472 vs. 19 sites) [134]. The RBD within the S protein is the primary target of neutralizing antibodies in naturally acquired or vaccine-elicited humoral immunity [135]. In our results, the RBD had a median WK of 4 with a maximum coefficient of 11.53 in site 484, followed by site 501, both located within the receptor-binding motif. Changes in these sites have been reported in several VOC variants and have been related to the neutralization escape of antibodies [136].

A similar variability increase was observed when comparing the N results to another study by the same author performed on global Nucleocapsid sequences (retrieved until July 2020), which showed lower variability [137]. The N protein presented a WK of 1 in 24% of the sites vs. 3.10% in our study, with only seven positions coinciding, together with more highly variable positions with a WK > 4 (64% vs. 27%) [137]. The N region with greater variability was the SR linker (WK 7), in line with previous global studies [126]. This region forms a phosphorylation-dependent binding domain for protein 14-3-3, a signaling molecule involved in various cellular processes, such as cell cycle, survival, and death [138].

On the other hand, Rahman et al.’s Wu–Kabat analysis of the Envelope, performed on global sequences retrieved until August 2020 [127], showed similar results to our analysis, finding the same percentage of E sites with a WK of 1 (16%) and almost the same number of variable sites with a WK > 4 (13 vs. 14 in our study). This suggests that, disregarding the geographical origin and the year of sampling, E is highly conserved among SARS-CoV-2 variants.

It has been reported that multiple introductions of SARS-CoV-2 to Spain took place at the beginning of the pandemic [5,112]. In the first study period (24 February to 20 June 2020), we detected 44 different lineages and sublineages circulating in Spain. In period 1.1, before the national lockdown, more than 60% of the Spanish sequences belonged to A lineages, in contrast to the predominance of B lineages found in other European countries during this period [139]. The two main A sublineages circulating in Spain at that moment were A.2 and A.5, both classified as endemic Spanish lineages [5,112]. However, B sublineages were more frequent in some AC (Figure 7), especially B.1, the second most frequent lineage after A.2 in our dataset. The B.1 lineage corresponds to a large European lineage whose origin is related to the Northern Italian outbreak early in 2020 [140], and it became the main circulating variant during the rest of period 1. The national lockdown effectively reduced the reproductive number and COVID-19 incidence [141,142]. The reduction in SARS-CoV-2 variants’ diversity between period 1.2 and period 1.3 suggests that the national lockdown was also effective in reducing the import of SARS-CoV-2 lineages during this period.

Two aa changes, D614G in the Spike protein and P323L in the RNA-dependent RNA polymerase (RdRp, nsp12), increased in frequency during period 1 and became dominant in the rest of the study periods (Figure 4). The success of the D614G mutation early in the pandemic was noted worldwide [126] and has been related to an increase in viral fitness [143,144]. This change was usually accompanied by RdRp P323L mutation, previously known as the “G clade” by GISAID nomenclature [144]. Although P323L is not located in the RdRp catalytic site, due to the RdRp’s key role in viral replication, any changes in its structure are of concern. It has been suggested that this change could alter RdRp’s interaction with its cofactors and anti-viral drugs [145]. Both changes have also been related to increased COVID-19 severity [146], but P323L’s effect on viral fitness remains unclear.

During period 2 (21 June to 5 December 2020), B.1.177 became the most successful lineage in Spain and the main lineage in most AC (9 AC in period 2.1 and 12 AC in period 2.2). This lineage has been related to the opening of borders within Europe during the summer of 2020, which allowed the rapid spread of the B.1.177 variant from Spain to other European countries [147]. Among the predominant aa changes detected during this period and related to this lineage (Figure 4), A222V Spike mutation had been detected in March in Tunisia and Iran, with a low mutation rate that increased in Spain in June 2020, similarly to A220V Nucleocapsid mutation [148]. In contrast to D614G, none of them have proved to confer increased transmissibility to the virus [147]. Therefore, the success of this lineage could be more directly linked to a lack of epidemiologic control in the viral spread than to an increase in viral fitness. The greater variant diversity detected in Spain during this period, most related to European countries but also from other continents, suggests that, despite the efforts to avoid SARS-CoV-2 spread between countries, travel restrictions during the summer of 2020 were not sufficient.

In the following periods, two VOC spread successfully after their introduction in Spain: Alpha and Delta. Both VOC have been associated with an increase in transmissibility and disease severity [149,150,151], but only the Delta VOC has shown evidence of an impact on immunity [152,153,154]. The Alpha VOC (B.1.1.7) was detected in our dataset for the first time in eight sequences collected in the Valencian Community in period 2.2 (October–November 2020). Its frequency increased during period 3 (December 2020–March 2021), representing almost half of the studied sequences, becoming the main circulating lineage in period 4 (March–June 2021). In the Spanish National Health report on circulating variants published on 26 March 2021, the Alpha variant represented >50% of the sequences in most AC and >70% in eight AC in the random sampling for epidemiological surveillance [155], becoming the dominant variant in June 2021 [156].

The Delta variant (B.1.617.2/AY) was detected for the first time in our dataset during period 4 in 11 AC, becoming the main circulating lineage of the following epidemic waves (June 2021–January 2022, periods 5 and 6). In the Spanish National Health report on circulating variants published in August 2021, the Delta variant increased its incidence during the summer of 2021, accounting for 47 to 96% of the COVID-19 cases across the different AC in July 2021 [157]. We found great diversity in the circulating Delta clusters detected in our sequence set. The ones detected during periods 4 and 5 were European, circulating mainly in Spain, the United Kingdom, Germany, and France. During the last period, Delta clusters common outside Europe, circulating in the United States of America, increased their frequency.

The rapid and efficient spread of these VOC suggests an increase in SARS-CoV-2’s viral fitness promoted by specific aa changes. In our analysis of the most frequent changes throughout the six epidemic waves (Figure 4), many of them were associated with certain periods in which one of the mentioned VOC prevailed. Spike mutations were the most abundant mutations, present in ≥10% of the total sequence dataset. Three of these mutations were located in the Spike RBD: L452R, T478K, and N501Y. L452R increased its frequency during periods 5–6. It is present in the Delta, Epsilon, and Kappa variants and has been related to immune escape [158,159]. T478K was mainly present in the last two periods. It can be found in the Delta and Omicron VOC and has been associated with increased ACE2 affinity and immune escape [160]. N501Y increased in periods 3–4 and 6. This aa change is present in the Alpha, Beta, Gamma, and Omicron VOCs, being Alpha the main lineage in period 4 and Omicron an increasing lineage in period 6. N501Y has been associated with greater ACE2 affinity and increased viral replication in human upper airway cells [161,162,163]. Several highly prevalent aa changes were located in the Spike’s S1 subunit (outside the RBD), five of them being deletions. H69del, V70del, and Y145del increased during periods 4 and 6. They are present in the Alpha and Omicron VOC. H69del and V70del have been associated with increased infectivity in Spike proteins that have acquired immune escape mutations that carry an infectivity cost [164,165]. Y145del has been described to impact immunity [166,167]. Deletions in sites 157–158, together with E156G (periods 5–6), have been associated with higher infectivity and reduced sensitivity to neutralization [152,158] and are present in the Delta VOC. The Spike site 618 is located next to the SARS-CoV-2 furin cleavage site. Two aa changes, P681H/R, were found in this site, mainly in periods 4 and 5, respectively. P681H is present in the Alpha and Omicron VOC and may increase the rate of Spike protein cleavage [163,168], although this mutation has not been proven to impact viral entry or spread [169]. P681R, also present in the Delta VOC, has been reported to have a similar effect as that described in P681H, increasing furin-mediated cleavage [170]. During period 4, A570D (end of S1 subunit) and S982A (S2 subunit) frequency increased. These mutations are present in the Alpha variant and may enhance cleavage into the S1 and S2 subunits by reducing the intermolecular stability of Spike protein subunits [163]. Fewer highly prevalent mutations were located in the Spike S2 subunit. Among them, D1118H (present in the Alpha variant) increased in period 4. This mutation has been suggested to impact trimer assembly [171], but its implications are not well known.

Another structural protein where several highly prevalent mutations could be found was the Nucleocapsid. Among these changes, three were located in the SR linker: R203K/M and G204R. R203K and G204R increased in period 4. It is a double aa change observed in global sequences [126]. It has been reported that these changes have arisen by homologous recombination rather than stepwise mutation and that viruses harboring these aa changes may also have increased expression of sub-genomic RNA from other open reading frames [172]. The Nucleocapsid protein’s main functions involve RNA binding, the replication and transcription of viral RNA, and the formation and maintenance of the ribonucleoprotein complex (see Table 1). However, it also participates in type I IFN inhibition [35,36] and the upregulation of subgenomic RNA and protein levels of the N protein have been observed in the Alpha variant, leading to enhanced immune evasion [173].

As for the other proteins, highly prevalent changes were found in non-structural proteins 3, 4, 6, 12, 13, and 14, and the accessory proteins ORF3a, ORF7a/b, and ORF 8. Most of these aa changes’ biological implications are not well known. However, nsp3, nsp6, ORF7a, and ORF 8 have been associated with host immune response evasion, as described in Table 1. The nsp6 deletions 106–108 are present in the Alpha, Beta, Gamma, Eta, Iota, Lambda, and Omicron variants, and could play a role in IFN-I evasion [161] due to nsp6’s role in antagonizing the type I interferon (IFN-I) response [43]. ORF8 has also been related to the modulation of the immune response (Table 1), and although the implications of R52I and Y73C are unknown, they may impact the ORF8 structure. R52 establishes two hydrogen bonds that stabilize its structure, and Y73 is part of a motif responsible for stabilizing an extensive noncovalent dimer interface [88]. ORF7a has been less studied, but this protein is involved in type I INF inhibition [43], NF-κB activation [80], JNK and IL-8 activation [80], and modulation of the inflammatory response (see Table 1). Further studies should be performed to clarify the impact of accessory protein mutations in SARS-CoV-2 host immune evasion. Nsp3 also plays an important role in other crucial functions such as polyprotein processing and viral spread (Table 1). Nsp12 (RNA-dependent RNA polymerase), 13 (Helicase), and 14 (Exonuclease) have major implications in the RTC (Table 1).

The Beta (B.1.351) and Gamma (P.1) VOC were also detected in Spain after period 3 but in low frequency (<1%). According to the official reports, these VOC were present in Spain in the following months but in a small proportion [156]. In our analysis, the Gamma VOC reached greater prevalence during period 4 (4.44%) and the Beta VOC during period 5 (1.61%).

The first sequences belonging to the Omicron VOC in our dataset were detected in the last period (October 2021-January 2022) in 12 AC, representing 26.99% of the Spanish sequences circulating in period 6. The Omicron variant was first reported to WHO from South Africa on 24 November 2021 and later declared a VOC. This variant is the most mutated SARS-CoV-2 variant to date and has been associated with an increase in infectivity and transmissibility and immune escape, but not with greater COVID-19 severity, and even with milder symptoms [124,174]. However, it exhibits significant resistance to the neutralizing activity of current vaccines [174,175]. According to the January 2022 Spanish National Health report, the Omicron variant was introduced into Spain in late November 2021, increasing its incidence progressively until it surpassed Delta in mid-December 2021, accounting for 70–90% of the cases in the different Spanish AC [176]. Among the Omicron sequences studied, 99.6% were BA.1 sublineages, with only 22 sequences belonging to the BA.2 sublineage. Both BA.1 and BA.2 are considered Omicron VOC, although they differ in their genetic sequence [108,174]. In a later report, published in February 2022, there was an increasing tendency in sublineage BA.2 cases [177]. As of May 2022, BA.2 is the predominant sublineage in Spain [178].

Spike mutations have been studied in more depth than other SARS-CoV-2 protein mutations, mainly due to the protein’s major role in infection, vaccine development, and antibody escape that some of these mutations may elicit [179,180,181]. However, it is essential to study nsp and accessory protein mutations and their implications, as many highly successful variants share mutations in proteins other than the Spike that could impact the host immune response or viral fitness. For this, randomized sequencing should be continued even in low-incidence settings. Moreover, SARS-CoV-2 sequencing should be encouraged in low-income countries by implementing international collaborations when possible.

To date, 75% of the European population has received at least one dose of COVID-19 vaccine, according to the ECDC. In Spain, the COVID-19 vaccination campaign began in December 2020 and was developed in stages, prioritizing certain population groups after the evaluation of their risk of exposure, transmission, and serious disease, as well as the socioeconomic impact of the pandemic, mainly healthcare workers and elders [182]. To date, 93% of the population over 12 years of age has received full vaccination and 80% of them at least one booster dose, while more than 40% of the pediatric population has received the complete vaccination schedule [182].

The Spike protein is the main protein used as a target in COVID-19 vaccines. Vaccine-induced neutralizing antibodies (nAbs) can target the S protein to inhibit virus infection at multiple stages during the virus entry process, being the RBD the major target for nAbs interfering with viral receptor binding [183,184]. Furthermore, the S protein is also a target for T-cell responses [185]. There has been some controversy regarding whether vaccination can be a source of SARS-CoV-2 mutations [186], and two antibody-disruptive co-mutations in the Spike (Y449S and N501Y) have been described as a new vaccine-resistant transmission pathway [187]. However, it has also been stated that vaccines can prevent their emergence [188,189]. SARS-CoV-2’s main mechanism of evolution is natural infectivity-based selection [187], where a high number of infections and high viral load within the host would facilitate the emergence of a wider range of mutations. Current vaccines have proven effective in reducing the number of infections and hospitalizations [190,191]. Even in the presence of VOC with mutations that alter vaccine efficacy, full vaccination is effective against severe COVID-19 caused by non-Omicron variants [192], resulting in a milder and shorter course of COVID-19, while booster doses have proven to improve neutralization against Omicron [175]. Furthermore, intra-host viral evolution during persistent infections leading to SARS-CoV-2 mutations identified in immune escape variants has been observed in immunocompromised patients [193,194]. In spring 2022, the mandatory use of face masks was repealed in Spain [195]. Although Omicron infections are generally milder, given the increasing incidence of COVID-19 in Spain, a second booster dose for elders and immunocompromised patients who are at risk of hospitalization should be considered. Meanwhile, the development of vaccines that include Omicron mutations should be encouraged. Currently, Pfizer and Moderna are evaluating Omicron-based vaccines [196,197].

However, emerging SARS-CoV-2 variants presenting a large number of mutations in the Spike protein may interfere with vaccine efficacy, as has been observed with the Omicron variant [174,175], and other targets should be considered for vaccine development. Within the Spike, according to our data, the S2 subunit is more conserved than the S1 subunit. S2 can also be a potential target for nAbs that interfere with the structural rearrangement of the S protein and the virus–host membrane fusion [198,199], and it would be interesting to include it in vaccine design together with other SARS-CoV-2 protein targets. However, this subunit contains more extensive N-glycan shielding and is less immunogenic than S1 [200].

According to our data, the E and M proteins are highly conserved among variants, which would make them suitable candidates for vaccine development. These proteins have already been proposed as vaccine targets [201,202]. However, the M and E proteins are poorly immunogenic [203], although they present T-cell epitopes in SARS-CoV and MERS-CoV [204]. Therefore, similarly to the Spike S2 subunit, these two proteins could be useful to broaden vaccine protection if included together with the S1 Spike subunit as an optimization strategy.

Another suitable option to avoid vaccine inefficacy due to emerging mutations is the use of inactivated or attenuated vaccines that contain the complete virus, which would theoretically induce broader antibody and T-cell responses, being less likely to become ineffective in the context of new SARS-CoV-2 variants. Currently, there is one live attenuated virus and nine inactivated virus vaccines in phases III and IV of clinical evaluation according to the WHO [205].

Nevertheless, considering currently available vaccines, a large part of the world population remains unvaccinated, with the global risk that this poses. For this reason, the WHO and other entities have created the Multilateral Leaders Task Force on COVID-19 Vaccines, Therapeutics, and Diagnostics (www.covid19taskforce.com (accessed on 12 May 2022)), whose aim is to vaccinate 40% of each country’s population by the end of 2021 and 60% by mid-2022, an aim that has yet to be met in most African countries. Access to vaccines in developing countries is a major concern, and European and other developed countries should promote these objectives to the best of their ability.

As for therapeutical approaches, at the beginning of the pandemic, the high mortality and lack of effective treatment options encouraged the use of repurposed drugs such as chloroquine or lopinavir [206,207], lacking robust clinical evidence of their efficacy and no longer recommended by the WHO [208]. Clinical trials are still under development for various monoclonal antibodies, although many have been ceased due to futility [209]. Remdesvir (Veklury by Gilead Sciences), a broad-spectrum antiviral originally developed to treat other viruses such as Ebola, was the first repurposed drug approved by the FDA for the treatment of hospitalized people aged 12 years and older with COVID-19 [210,211]. This drug inhibits viral RNA-dependent RNA polymerase (RdRp, nsp12) while evading proofreading by viral exoribonuclease, which leads to premature termination of RNA transcription [212]. An initial WHO conditional recommendation made in November 2020 suggested not to use remdesivir for patients with COVID-19, regardless of illness severity. However, in the tenth iteration of the guideline, a new WHO recommendation was made for the use of remdesivir for patients with non-severe illness at highest risk of hospitalization [208]. The recommendation for patients with severe or critical COVID-19 is currently under review and it will be updated shortly. Using computational approaches, it has been proposed that remdesivir binds to more than one target of SARS-CoV-2, showing strong binding affinity with the M protein, RdRp, and np5 or 3CLpro [110].

Regarding new drugs to be developed, non-structural proteins are good candidates considering their lower Mf and highest conservation according to our data, together with their critical role in the replication and transcription complex (Table 1). Among them, some proteins can be interesting drug targets, such as the previously mentioned 3-chymotrypsin-like protease (3CLpro or nsp5), the protein with the lowest Mf in our dataset (7.73 × 10^−6^), involved in polyprotein processing (see Table 1). Indeed, 3CLpro inhibitor molecules have proven to increase survival in infected mice [213] and have been considered as candidates to inhibit SARS-CoV-2 [214].

Currently, an elective inhibitor of the SARS-CoV-2 3CLpro developed by Pfizer (paxlovid, nirmatrelvir-ritonavir) has reached a phase III trial [209], and the WHO has set a strong recommendation for its use in non-severe COVID-19 patients at highest risk of hospitalization, considering it the best therapeutic choice for high-risk patients to date [215]. Pfizer’s oral antiviral drug paxlovid (PF-07321332 + ritonavir) reduces hospital admissions and deaths among people with COVID-19 who are at high risk of severe illness (with a reported reduction of 89% within three days of symptom initiation) when compared with a placebo [214,216]. PF-07321332 is a reversible covalent inhibitor that targets SARS-CoV-2 3CL-pro, forming a covalent bond to the catalytic nsp5 residue C145, being further stabilized through a network of hydrogen bonds and hydrophobic interactions, which enhance its binding to the active site of 3CL-pro, involving another five residues [111]. In our variability analysis of the sites involving paxlovid and remdesivir binding, we found high conservation in all the residues. No aa changes were detected in C145 in our dataset, and only a single sequence presented a deletion in one of the residues (Q192) that enhanced nsp5 binding to paxlovid. However, future analysis should be conducted to survey the emergence of 3CL-pro mutations in the context of SARS-CoV-2 treatment regarding these drugs.

Other nsp proteins that were highly conserved in our results were the helicase (nsp13), with the third lowest Mf (Table 2), and the 2′-O-Methyltransferase (nsp16). However, many factors, including toxicity, bioavailability, and effective delivery, must be considered for drug development. Clinical trials are complex and expensive, and the fall in mortality due to vaccination, higher preparedness in hospitals, and preventive measures such as face masks and hand-washing may reduce the efforts devoted to the development of new drugs against COVID-19. Nevertheless, similarly to new vaccine development, in the context of the increasing SARS-CoV-2 variability detected in many of its proteins in this study, the continuous emergence of new variants across the globe, and the risk of the future reemergence of this virus or other coronaviruses, drug development should be encouraged and pursued.

The main limitation of this study is the uneven number of SARS-CoV-2 sequences across AC and study periods available in the GISAID database, especially at the beginning of the pandemic. This was due to many factors, such as technical and economic availability for SARS-CoV-2 sequencing across the Spanish hospitals, variable incidence among periods and AC, and differences in the diagnostic protocols between AC.

Although the present study only focuses on the SARS-CoV-2 evolution in one country during the first year of the pandemic, these data can be of high interest since Spain has been one of the main epicenters for COVID-19, reaching the highest number of cases and deaths per 100,000 population in Europe at the beginning of the pandemic. Furthermore, the fact that Spain is one of the leading European tourist destinations can favor the spread of new SARS-CoV-2 variants and could explain the high diversity of circulating variants observed in our study, mainly after the lockdown.

## 4. Materials and Methods

SARS-CoV-2 sequences were downloaded in nucleotides (nt) from the publicly available GISAID repository (https://www.gisaid.org/ (accessed on 02 February 2022)). We selected those sequences classified as human hosts, located within Europe/Spain and ascribed to an Autonomous Community (AC), submitted until 2 February 2022, and collected from 24 February 2020 to 29 January 2022. We then classified the sequences according to the epidemiological week (epiweek) by collection date. Epiweeks are a standardized method of counting weeks to allow for the comparison of epidemiological data. By definition, the first epiweek of the year ends on the first Saturday of January, as long as it falls at least four days into the month. Each epiweek begins on a Sunday and ends on a Saturday. The present study included SARS-CoV-2 sequences collected from 2020 epiweek 9 (24 February 2020) to 2022 epiweek 4 (29 January 2022).

To contextualize the changes in the virus throughout the pandemic, epiweeks were grouped into six main periods adjusted to the Spanish epidemic curve, as informed by the National Epidemiological Surveillance Network (RENAVE) [217]. Period 1 was further divided into three phases according to the Spanish government’s measures implemented to prevent the spread of the virus: period 1.1 before the national lockdown, period 1.2 during the national lockdown until the beginning of the national deconfinement plan, and period 1.3 until the end of the first epidemic wave. Period 2 was subdivided into two periods according to the two peaks of incidence in this second epidemic wave, one after summer 2020 with a rise in the instantaneous basic reproductive number (Rt) at the beginning of July included in period 2.1, and a second peak before winter 2020 with another rise in the Rt in mid-October covered by period 2.2. The time span and major events of each study period are described in Table 4.

Wuhan SARS-CoV-2 was taken as the reference sequence (NCBI accession number NC 045512.2) to identify the nt mutations and aa changes in the annotated proteins. Sequence analysis was performed with an in-house bioinformatics tool (EpiMolBio) previously designed and used in our laboratory for HIV genetic variability analysis and recently updated for SARS-CoV-2 sequence study [126,218,219,220,221,222]. This tool is programmed in JAVA OpenJDK version 11.0.9.1 using IDE NetBeans version 12.2 and allows the simultaneous analysis of a high number (>650,000) of sequences. Functions related to protein tracking, trimming, and aligning were tested with Mega X, and functions related to aa change identification were tested manually and using Excel 2019 version 19.0. Using EpiMolBio tool, the complete nt sequences from 26 structural, non-structural (nsp), and accessory viral proteins were cut, aligned, and translated into amino acids (aa). The final analysis included nsp1–10 (polyprotein1a and 1ab), nsp11 (polyprotein 1a), nsp12–16 (polyprotein1ab), structural proteins Spike (S), Nucleocapsid (N), Membrane (M), and Envelope (E), and accessory proteins 3–10, according to NCBI 045512.2 annotation. This program detects any nt/aa in the sequence set different to the reference one for each position and calculates the number and frequency of nt/aa changes for that site, ignoring unidentified nt, nonsense mutations, and unknown amino acids that could be present due to the low quality of some regions of the original sequences, failing to attribute a nucleotide with certainty. EpiMolBio tool allows the analysis of partial or low-quality genomes as long as the residue of the studied position is present, enabling a much larger set of sequences to be studied.

The number of polymorphisms in the SARS-CoV-2 pan-genome and each studied protein was calculated, as well as the ratio of transitions (nt changes between the two purines A and G or between the two pyrimidines C and T) and transversions (nt changes between a purine and a pyrimidine). We also calculated the frequency of base mutations (Mf) or mutation frequency according to the following formula: Mf = P i/(Ln × N) [121], being Pi the number of instances of polymorphism detected, Ln the nucleotide length of the genome or locus, and N the number of sequenced entities present in the dataset.

In each of the 26 SARS-CoV-2 proteins, we calculated and compared the mean aa conservation, the number of aa changes, deletions, or stops, and the number of conserved and variable positions within each protein. We also identified the presence of mutations and the variability of the SARS-CoV-2 main protease (nsp5 or 3CL-pro) residues involved in binding with two of the current WHO-recommended drugs for COVID-19 [208], remdesivir and nirmatrelvir-ritonavir (sold under the name Paxlovid).

The Wu–Kabat protein variability coefficient (WK) was calculated and analyzed in the context of the proteins’ domains and relevant functional sites, according to the Uniprot database (https://www.uniprot.org (accessed on 15 February 2022)) annotation. This coefficient allows the study of the susceptibility of an aa position to evolutionary replacements [223]. It is calculated using the following formula: Variability = N × k/n, where N is the number of sequences in the alignment, k is the number of different amino acids at a given position, and n is the absolute frequency of the most common amino acid at that position. Therefore, a WK of 1 indicates that the same aa was found for that position in the entire sequence set, whereas a WK ˃ 1 indicates the relative variability of the respective site, with greater diversity as the WK value increases.

The frequency of the aa changes and deletions was calculated in all the Spanish sequences. Those changes present in ≥10% of the sequences were further studied. To detect the behavior of these changes in time (increase or decrease in their frequency), they were analyzed in the six main periods previously described, calculating the frequency difference between periods (Δ) and comparing it. A second analysis was carried out considering the aa changes and deletions different to those previously detected in the complete Spanish dataset and present in ≥10% of each of the 17 AC and two Autonomous Cities. This allowed us to detect any relevant aa change limited to a particular AC, given that the course of the pandemic and the containment measures established since the deconfinement plan differed between AC. The changes were located in each protein, compared between AC, and those with a high prevalence (≥25%) were also analyzed by period. The 17 Spanish AC are Andalusia, Aragon, Asturias, the Balearic Islands, Basque Country, the Canary Islands, Cantabria, Castile La Mancha, Castile and Leon, Catalonia, Extremadura, Galicia, La Rioja, Madrid, Murcia, Navarre, and the Valencian Community. The two Autonomous Cities were grouped into an 18th AC to simplify the analysis comprehension; these are Ceuta and Melilla, both located in the North of Africa.

For the correct assignment of the SARS-CoV-2 variants, the quality of all the downloaded sequences was checked using Nextclade v.1.11.0 (https://clades.nextstrain.org/ (accessed on 10 May 2022)), and the sequences classified as “bad” quality were removed from the analysis. This tool performs quality control based on a score that considers missing data, mixed sites, private mutations, mutation clusters, stop codons, and frameshifts. The remaining sequences were assigned to the genetic lineages according to Pangolin COVID-19 Lineage Assigner v 4.0.6 (https://pangolin.cog-uk.io/ (accessed on 10 May 2022)) to contextualize the aa changes found in the different phases and the evolution of the pandemic in Spain during the study period. For this analysis, we also included 417 additional sequences from the Canary Islands that had not been classified according to the location criteria previously described in this section after confirming their geographical origin. The Pangolin COVID-19 Lineage Assigner software assigns lineages using a nomenclature based on a hierarchical system [104] and is the one currently used in Spain for the epidemiological monitoring of COVID-19. The Pangolin lineage list (https://cov-lineages.org/lineage_list.html (accessed on 10 May 2022)) was used to locate the main countries of origin of the detected Spanish lineages.

## Figures and Tables

**Table 1:** Proposed molecular functions of the twenty-six SARS-CoV-2 proteins.

Protein	Proposed Molecular Function
**I. Structural proteins**	
Spike (S)	Class I fusion protein that mediates attachment to the host cell’s receptor angiotensin-converting enzyme 2 (ACE2) through the receptor-binding domain (RBD), and fusion of viral and cellular membranes [21,22,23]
Envelope (E)	Viral assembly and release through interaction with M protein [24,25,26,27], epithelial cells’ tight junctions’ disruption by interaction with PALS1 [28,29].
Membrane (M)	Virion shape, participates in E assembly and N attachment to the viral genome, interacts with S [30,31,32].
Nucleocapsid (N)	Nucleocapsid protein, binding to RNA genome, participates in transcription and replication, interaction with M during viral assembly [27,30,33,34], type I IFN inhibition [35,36].
**II. Nonstructural proteins**	
nsp1	Leader protein, suppresses host gene expression by ribosome association, mediates RNA replication [37,38,39,40,41], type I IFN inhibition [35,37,42,43].
nsp2	Related to the disruption of intracellular host signaling in SARS-CoV infections [44].
nsp3	Papain-like protease [45,46], polyprotein processing [47]. Type I IFN inhibition [35,46], implicated in membrane structure formation that is induced upon CoV infection and with which the RTC is thought to be associated [48,49,50].
nsp4	Implicated in membrane structure formation that is induced upon CoV infection and with which the RTC is thought to be associated [48,49].
nsp5	Chymotrypsin-like protease (3CLpro) (main protease), polyprotein processing [51,52].
nsp6	Induction of autophagosomes and limit of autophagosome expansion [53]. INF inhibition [43], implicated in membrane structure formation that is induced upon CoV infection and with which the RTC is thought to be associated [48].
nsp7	Processivity cofactor for RdRp [54,55].
nsp8	Processivity cofactor for RdRp [54,55].
nsp9	Single-strand nucleic acid-binding protein [56,57]. Possibly involved in the capping process: nsp9 may inhibit nsp12 NiRAN GTase activity in an intermediate state of RTC for further cap structure synthesis [58].
nsp10	Increases nsp14 exoribonuclease and nsp16 2′-O-methyltransferase activities [54,59,60,61].
nsp11	Unknown
nsp12	RNA-dependent RNA polymerase (RdRp), replication and transcription of the viral RNA genome [62,63,64], type I IFN inhibition [35].
nsp13	Superfamily 1 helicase with a zinc-binding domain involved in RTC: participates in capping [58], unwinds RNA duplexes with 5′ to 3′ direction [65,66,67], and has 5’ triphosphophatase activity [68]. Type I INF inhibition [35,43,69].
nsp14	Proofreading exoribonuclease and N7 guanine-methyl transferase activity involved in the viral mRNA cap synthesis [70,71,72,73,74].
nsp15	Uridylate-specific endoribonuclease activity [75], may counteract double-strand RNA sensing [76]. Type I INF inhibition [69].
nsp16	2′-O-Methyltransferase: mRNAs cap 2′-O-ribose methylation to the 5′-cap structure [60,77,78].
**III. Accessory proteins**	
3a	Type I INF inhibition [43], virulence [79], NF-κB activation [80,81], JNK and IL-8 activation [80], ion-channel activity [81], enhanced production of inflammatory chemokines [80], apoptosis induction, and necrosis [82,83].
6	Type I INF inhibition [35,36,43,69], enhances viral replication [84], virulence [79].
7a	Type I INF inhibition [43], NF-κB activation [80], JNK and IL-8 activation [80], modulation of the inflammatory response [85].
7b	Unknown
8	Type I INF inhibition [36], mediates immune evasion [86,87,88] and inflammation [89], interacts with proteins involved in ER protein quality control and ubiquitin-dependent endoplasmic reticulum-associated degradation pathways [90,91].
10	There is controversy regarding its expression and whether it is a coding protein [92,93]. May affect the immune response [94,95].

**Table 2:** Polymorphisms, transitions and transversions ratio, and mutation frequency detected in Spanish SARS-CoV-2 sequences during the first two years of the pandemic among the 26 viral proteins.

Locus	Number of Sequences	Location	Length (bp)	Number of Polymorphisms	Ts:Tv Ratio	Mean Mutation Frequency
nsp1	86,080	266–805	540	621	1:0.64	1.34 × 10^−5^
nsp2	85,659	806–2719	1914	2446	1:0.87	1.49 × 10^−5^
nsp3	83,819	2720–8554	5835	7310	1:0.98	1.49 × 10^−5^
nsp4	84,434	8555–10,054	1500	1130	1:0.49	8.92 × 10^−6^
nsp5	85,208	10,055–10,972	918	605	1:0.44	7.73 × 10^−6^
nsp6	85,511	10,973–11,842	870	777	1:0.73	1.04 × 10^−5^
nsp7	86,668	11,843–12,091	249	257	1:0.78	1.19 × 10^−5^
nsp8	86,849	12,092–12,685	594	405	1:0.43	7.85 × 10^−6^
nsp9	86,713	12,686–13,024	339	262	1:0.45	8.91 × 10^−6^
nsp10	84,592	13,025–13,441	417	290	1:0.51	8.22 × 10^−6^
nsp11	84,593	13,442–13,480	39	39	1:1.29	1.18 × 10^−5^
nsp12	84,069	13,442–16,236	2796	2934	1:1	1.25 × 10^−5^
nsp13	85,477	16,237–18,039	1803	1212	1:0.49	7.86 × 10^−6^
nsp14	84,666	18,040–19,620	1581	1210	1:0.50	9.04 × 10^−6^
nsp15	85,788	19,621–20,658	1038	1021	1:0.78	1.15 × 10^−5^
nsp16	85,050	20,659–21,552	894	651	1:0.65	8.56 × 10^−6^
gene S	83,928	21,563–25,384	3819	5486	1:1.28	1.71 × 10^−5^
ORF3a	86,034	25,393–26,220	825	1055	1:0.90	1.49 × 10^−5^
gene E	85,937	26,245–26,472	225	234	1:0.92	1.21 × 10^−5^
gene M	85,720	26,523–27,191	666	522	1:0.65	9.14 × 10^−6^
ORF6	85,701	27,202–27,387	183	194	1:0.81	1.24 × 10^−5^
ORF7a	82,217	27,394–27,759	363	621	1:1.16	2.08 × 10^−5^
ORF7b	82,083	27,756–27,887	129	133	1:0.82	1.26 × 10^−5^
ORF8	84,992	27,894–28,259	363	513	1:0.92	1.66 × 10^−5^
gene N	70,124	28,274–29,533	1257	2277	1:1.49	2.58 × 10^−5^
ORF10	82,312	29,558–29,674	114	129	1:0.55	1.37 × 10^−5^
Complete Genome				32,334	1:0.90	1.24 × 10^−5^
Non-structural proteins				21,170	1:0.78	1.05 × 10^−5^
Structural proteins				8519	1:2.26	1.60 × 10^−5^
Accessory proteins				2645	1:0.93	1.52 × 10^−5^

Genes located according to reference SARS-CoV-2 sequence NCBI 045512.2. bp: base pair; Ts: transition; Tv: transversion. S: Spike; E: Envelope; M: Membrane; N: Nucleocapsid; nsp: non-structural protein.

**Table 3:** Number of aa changes, deletions, stop codons, percentage of variable aa positions, and conservation across Spanish SARS-CoV-2 sequences in each of the 26 viral proteins.

Protein	Number of Sequences	Length (aa)	Number of Changes (aa; Deletions; Stops)	Mean Changes per Sequence *	Variable Positions (%)	aa Conservation (%)
nsp1	86,080	180	438 (404; 32; 2)	0.10	92.78	99.95
nsp2	85,659	638	1614 (1545; 48; 21)	0.41	93.73	99.94
nsp3	83,819	1945	4921 (4364; 423; 134)	3.22	91.36	99.83
nsp4	84,434	500	671 (661; 4; 6)	1.15	72.80	99.77
nsp5	85,208	306	334 (322; 9; 3)	0.16	64.71	99.95
nsp6	85,511	290	514 (471; 35; 8)	1.79	81.03	99.38
nsp7	86,668	83	144 (129; 7; 8)	0.02	90.36	99.98
nsp8	86,849	198	237 (236; 0; 1)	0.03	74.75	99.98
nsp9	86,713	113	146 (139; 3; 4)	0.03	72.57	99.97
nsp10	84,592	139	154 (152; 1; 1)	0.02	64.03	99.98
nsp11	84,593	13	20 (20; 0; 0)	0.00	76.92	99.97
nsp12	84,069	932	1832 (1526; 207; 99)	1.88	87.34	99.80
nsp13	85,477	601	648 (638; 1; 9)	0.69	63.73	99.89
nsp14	84,666	527	734 (704; 19; 11)	0.72	71.16	99.86
nsp15	85,788	346	659 (600; 39; 20)	0.09	85.26	99.98
nsp16	85,050	298	385 (371; 8; 6)	0.07	72.15	99.98
S	83,928	1273	3838 (3318; 397; 123)	10.80	91.52	99.13
ORF3a	86,034	275	811 (736; 59; 16)	0.87	95.64	99.68
E	85,937	75	150 (133; 10; 7)	0.12	85.33	99.84
M	85,720	222	288 (281; 2; 5)	0.78	69.82	99.64
ORF6	85,701	61	138 (123; 6; 9)	0.02	95.08	99.97
ORF7a	82,217	121	499 (408; 62; 29)	1.08	99.17	99.10
ORF7b	82,083	43	105 (92; 8; 5)	0.45	97.67	98.96
ORF8	84,992	121	396 (338; 27; 31)	1.60	100.00	98.67
N	70,124	419	1661 (1459; 170; 32)	3.79	99.28	99.09
ORF10	82,312	38	96 (91; 2; 3)	0.09	97.37	99.77
Complete genome		9757	21,433 (19,261; 1579; 593)	1.15	84.06	99.69
Non-structural proteins		7109	13,451 (12,282; 836; 333)	1.25	79.19	99.84
Structural proteins		1989	5937 (5191; 579; 167)	3.87	86.49	99.42
Accessory proteins		659	2045 (1788; 164; 93)	0.68	97.49	99.36

Conserved positions included all protein residues without any aa change, stop codon, or deletion; aa: amino acid; del: deletions; %: percentage; nsp: non-structural protein. * including aa changes and deletions.

**Table 4:** Study periods included in this study and relevant events.

Periods	Epiweeks	Dates	Relevant Events
Period 1	09.2020 to 25.2020	24 February 2020 to 20 June 2020	First Spanish COVID-19 wave. First state of emergency.
1.1	09.2020 to 11.2020	24 February 2020 to 14 March 2020	From the beginning of the pandemic until the national lockdown (15 March 2020).
1.2	12.2020 to 18.2020	15 March 2020 to 02 May 2020	From the national lockdown until the beginning of the national deconfinement plan.
1.3	19.2020 to 25.2020	03 May 2020 to 20 June 2020	End of the first epidemic wave.
Period 2	26.2020 to 49.2020	21 June 2020 to 05 December 2020	Second COVID-19 Spanish wave.
2.1	26.2020 to 40.2020	21 June 2020 to 03 October 2020	First peak of incidence after 2020 summer with a rise in the R_t_* on early July.
2.2	41.2020 to 49.2020	04 October 2020 to 05 December 2020	Second peak of incidence before 2020 winter with another rise in the R_t_ in mid-October. Second state of emergency and beginning of the third state of emergency.
Period 3	50.2020 to 10.2021	06 December 2020 to 13 March 2021	Third Spanish epidemic wave. Introduction of B.1.1.7 or Alpha variant. Start of the COVID-19 vaccination campaign.
Period 4	11.2021 to 24.2021	14 March 2021 to 19 June 2021	Fourth Spanish epidemic wave. Alpha became the main circulating variant in Spain. Introduction of Delta variant during the last half of the period. End of the third state of emergency in May.
Period 5	25.2021 to 41.2021	20 June 2021 to 16 October 2021	Fifth Spanish epidemic wave. Delta became the main circulating variant in Spain.
Period 6	42.2021 to 04.2022	17 October 2021 to 29 January 2022	Sixth Spanish epidemic wave. Introduction of the Omicron variant, which quickly became the main circulating variant in Spain.

* basic reproductive number.

## Data Availability

Sequences publicly available in the GISAID database https://www.gisaid.org (accessed on 2 February 2022).

## Supplementary Materials

The following supporting information can be downloaded at: https://www.mdpi.com/article/10.3390/ijms23126394/s1.
