# Supplementary figures and images for: Evolution of SARS-CoV-2 in Spain during the First Two Years of the Pandemic: Circulating Variants, Amino Acid Conservation, and Genetic Variability in Structural, Non-Structural, and Accessory Proteins
Source: Int J Mol Sci. 2022 Jun 7;23(12):6394. doi: 10.3390/ijms23126394 (PMC9223475; doi:10.3390/ijms23126394)

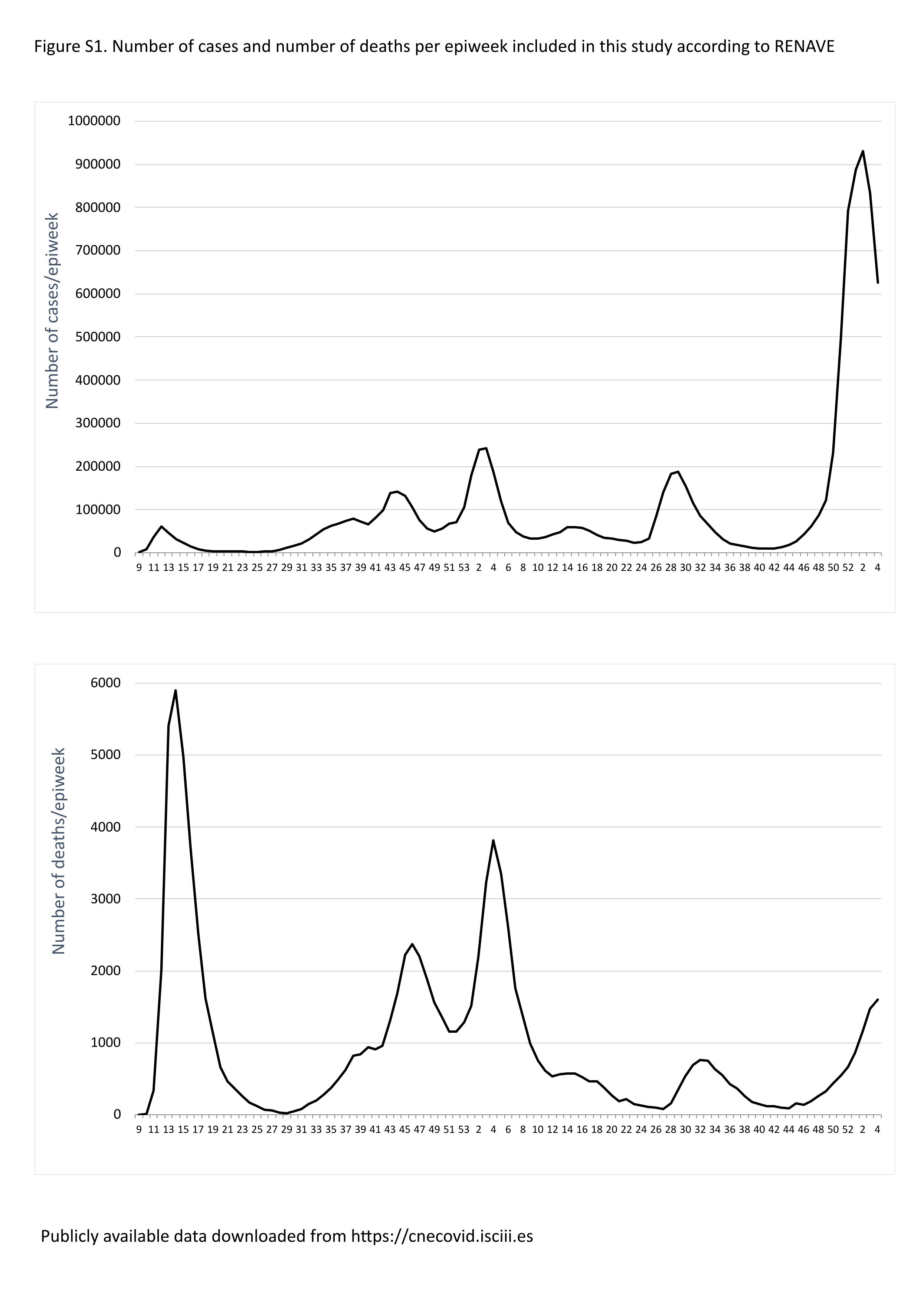

Supplement: Supplementary file 1 [file ijms-23-06394-s001.zip › Sup/Figure S1.png]
